# System Action Learning: Reorientating Practice for System Change in Preventive Health

**DOI:** 10.1007/s11213-023-09638-y

**Published:** 2023-03-30

**Authors:** Therese Riley, Liza Hopkins, Maria Gomez, Seanna Davidson, Jessica Jacob

**Affiliations:** 1 The Australian Prevention Partnership Centre, The Sax Institute, Sydney, Australia; 2 McMaster University, Hamilton, Canada

**Keywords:** System Action Learning, Chronic Disease Prevention, Systems Thinking

## Abstract

It is now widely accepted that many of the problems we face in public health are complex, from chronic disease to COVID-19. To grapple with such complexity, researchers have turned to both complexity science and systems thinking to better understand the problems and their context. Less work, however, has focused on the nature of complex solutions, or intervention design, when tackling complex problems. This paper explores the nature of system intervention design through case illustrations of system action learning from a large systems level chronic disease prevention study in Australia. The research team worked with community partners in the design and implementation of a process of system action learning designed to reflect on existing initiatives and to reorient practice towards responses informed by system level insights and action. We were able to observe and document changes in the mental models and actions of practitioners and in doing so shine a light on what may be possible once we turn our attention to the nature and practice of system interventions.

## Background

Nearly half of all Australians have one or more chronic conditions, such as cardiovascular disease and chronic obstructive pulmonary disease (Australian Bureau of Statistics 2018). Many of these are preventable. Prevention efforts have traditionally focused on lifestyle programs designed to reduce chronic disease risk factors such as unhealthy eating or harmful use of alcohol. However, such approaches are not yielding the gains needed at a population level and calls to take a radically different approach are mounting (Carey et al. 2015; Rutter et al. 2017). Over the past decade public health scholars have been calling for a shift away from reductionist and linear approaches to understanding public health problems and their solutions (Hawe et al. 2004; Hawe 2015a; Plsek and Greenhalgh 2001). It is now believed that embracing the complexity of chronic disease and looking to system science as a new worldview and methodology may get us closer to reversing trends such as obesity which contribute to the burden of chronic disease (Rusoja et al. 2018; Rutter et al. 2017).

The use of systems thinking methods and tools in public health research is also increasing, ranging from the creation of causal loop diagrams exploring obesity (Allender et al. 2015) through to dynamic agent based modeling which sheds light on efforts to reduce alcohol harm (Atkinson et al. 2018). A recent review of complex systems approaches to public health evaluations identified 74 studies that applied a range of methods highlighting the growth in their application. The authors of the review conclude that in order for the field to progress, methodological innovation will be necessary (McGill et al. 2021). This surge in systems evaluation, however, is not matched by interest in the scholarship of system interventions. This lack of attention to system intervention design and implementation means that many system evaluations are studying multifaceted programs that are not necessarily designed for systemic change. Hawe (2015a) argues that attention needs to be paid to intervention designs that genuinely grapple with complexity, otherwise we are destined to only see modest or ‘negligible’ gains for the health of populations (Hawe 2015b).

While there is a paucity of literature regarding the design of system interventions, there are some notable exceptions, particularly in the field of obesity prevention (Allender et al. 2021; Garcia et al. 2021). Interventions described in these studies are designed ‘with’ communities. They build on and strengthen pre-existing work in community capacity building, and involve action learning and systems thinking (Allender et al. 2021; Garcia et al. 2021). This interdisciplinary approach applies systems methods, creates coordinated actions across systems, expects and encourages adaptation and retains a focus on system behavior. At the heart of these interventions are ‘systems practices’ which are designed to shift mental models through learning and action. It is this combination that ensures intervention implementation is dynamic and able to cope with uncertainty. However, embedding a systems mindset across a workforce is not easy. In a study of the Healthy Together Victoria initiative in Australia (2011–2015) Bensberg (2021) found that the introduction of various systems theories, frameworks and methods to a health promotion workforce, did not necessarily translate into easily articulated practical examples of systems change (Bensberg 2021). While systems practices were evident in the initiative (Roussy et al. 2020), many practitioners struggled to describe the connection between systems ideas and their day-to-day work (Bensberg 2021). This suggests that more work is needed to bridge the gap between systems theory (methods and concepts) and practice.

Bringing together the tradition of action research and system science has led to the development of system action learning as a way of placing systems thinking in the action orientated contexts of practice. The purpose of action learning is to engage with real-life problems that lack a clear solution. It is a group process of learning and reflection that can produce further system insights and encourage actions which target systems-level changes (Zuber-Skerritt 2002). This process of participatory systemic inquiry fosters a ‘learning architecture’ where multiple inquiries can operate at once, allowing stakeholders to engage in action within and across various parts of the system. The ‘learning architecture’ links these inquiries together creating a space for stakeholders to effect systems change in meaningful ways (Burns 2014).

System action learning programs draw on a range of both action learning and systems traditions including Critical Systems Theory (CST) (Aragon and Giles Macedo 2010; Barta et al. 2016; Lewis 2015). Facilitated action learning processes that question assumptions, boundaries, and power dynamics, can create learning opportunities to generate knowledge about a system that otherwise may have not been explored. In doing so, actions and decisions derived from the system, are privileged and become/inform applied practice.

Learning systems foster an environment of cooperative learning whereby iterative learning processes are embedded in organisational or community culture and practice (Ison et al. 2007; Aragon and Giles Macedo 2010). Embedding learning processes is also at the heart of systems approaches such as Soft Systems Methodologies (SSM) that highlight the importance of cyclic inquiry (Checkland and Poulter 2010). Systemic learning is not a one-off event! The cyclic nature of these approaches is consistent with continuous quality improvement and the PDSA (Plan Do Study Act) cycle popular in health care (Taylor et al. 2014). Overall, there are a range of approaches, resources and tools within system action learning (Foster-﻿Fishman and Watso﻿n 2012; Wadsworth 2010) that could support and sustain positive change. For the purposes of this paper, we are focused on community based prevention efforts, rather than hospital or health care sectors.

## The Study Context

Prevention Tracker was a national initiative of the Australian Prevention Partnership Centre (Wilson et al. 2014). It was designed to better understand the nature of prevention efforts in local communities using a systems thinking approach. We worked with four geographically diverse communities across Australia to describe, guide and monitor change efforts in the chronic disease prevention systems. The work occurred across four domains of enquiry: describing a chronic disease prevention system; guiding system change; monitoring system change; and a cross-case comparison (Riley et al. 2020). Community in this study refers to geographic places defined by government boundaries.

Prevention Tracker was an ambitious and complex research intervention, which utilised numerous data sets and a multitude of systems thinking tools and approaches. It aimed to trial a new way of developing solutions to complex, systemic problems within chronic disease prevention - a field characterized by diffuse governance, siloed organisations, multiple funding sources and a focus on programmatic interventions (Del Fabbro et al. 2016, Thompson et al. 2015).

The full systems methodology of Prevention Tracker, including an outline of our approach to system action learning is described elsewhere (Riley et al. 2020). This paper examines the systems action learning component of Prevention Tracker which was part of the guiding and monitoring systems change domains. The system action learning activities were developed following the identification of a systemic problem in each of the communities and focused on projects already planned or being implemented within the community. System action learning took place in two of the four communities.

## Method

System action learning took the following form in Prevention Tracker. We worked with project partners in communities to identify three local projects which offered scope to incorporate a systems-action learning approach. These were prevention initiatives that were underway at the time and which the research team and the project partners jointly identified as ones where a systemic intervention may yield positive results.

This phase of Prevention Tracker involved facilitation of an iterative learning process whereby system-level insights could influence day-to-day prevention activities. Through the process, these activities can allow participants to surface additional system insights which provided feedback into their prior understanding of the system and in turn directed action to address system change. We present the system action learning associated with each project as three cases, each made up of a practice focus, 2–3 community team members and cycles of system action learning. Case 2 and 3 are made up of the same community team.

The cases were.

1. The functioning of a coalition of community organisations associated with health and wellbeing.

The local community team, made up of 3 government practitioners were grappling with how to maximise the role and impact of a community coalition of organisations providing information, advice and support to government. The system action learning cycles provided an opportunity to reflect on and reconsider the role of the coalition. The community was an urban local government area.

2. The delivery of a healthy eating initiative.

The local community team, made up of two practitioners, were involved in the implementation of a healthy eating initiative. They were grappling with how to involve more non traditional actors, such as the private sector. The system action learning cycles provided an opportunity to better understand the situation, surface assumptions and identify new practices. The community was a regional local government area.

3. The evaluation of a new health partnership between a local government and a state government organisation.

The local community team made up of two practitioners, were involved in the evaluation of the partnership. The system action learning cycles enabled the team to gain new system insights into the partnership and practices to evaluate impact. The community was a regional local government area.

System Action Learning (SAL) within Prevention Tracker involved an iterative cycle of the local community team undertaking their usual planning meeting for each project, then the completion of a Prevention Tracker reflection template to capture the operations of the meeting and the agreed actions which resulted. The completed template was forwarded to the Prevention Tracker research team, and used to inform the design of a tailored SAL activity. The SAL activity was then conducted by at least two members of the research team, together with the project partners in the community. This activity was usually undertaken in person for the first instance and then by telephone for subsequent cycles. The activity involved a 1.5- 2 hr facilitated sessions where a systems thinking specialist member of the research team led the local community team through a series of questions and answers. The process was designed to enable the project partners in the community to gain new and additional insights into the system within which they were working, and the local systemic problem they were trying to address.

The facilitated systems activities were each audio-recorded and transcribed verbatim. The final step in the SAL cycle involved the project partners completing a second template designed to help them reflect on and better understand their own role in the system. The completed template was again forwarded to the research team to help inform the study as to how the partners were understanding and learning from the process. The community project teams in each community went through 3 or 4 cycles of System Action Learning per project.

A total of 10 rounds of system action learning were undertaken (3 in two of the projects and 4 in the other project). Templates were filled out and systems activity sessions were audio recorded with consent. All data was imported into an NVIVO database (QSR 2012). Table 1 describes the codebook that was developed over a number of weeks to code this diverse data. We started with theoretically informed high level codes and then refined the codes and sub codes through a series of coding workshops (Crabtree and Miller 1999). Three researchers (LH, MG, TR) all coded a selection of transcripts and talked through the similarities and differences to refine the interpretation presented in the codebook. Once all researchers were comfortable and confident in the coding and interpretation, the entire data set was coded. Some codes were easier to apply and interpret than others.

**Table 1:** Codebook for System Action Learning

Code	Sub code
Action	
	Intention to act
	Action undertaken (By SAL Team)
	Action Undertaken (by others)
	Relational action
System Insights	
	Leverage Points
	Reflection on boundaries
	Reflection on relationships
	Reflection on different perspectives or view points
	System patterns (dynamics of the system)
	Reflection on system parts
	Unintended consequences, challenges or barriers
Double Loop Learning	
	Surfacing assumptions
	Challenging assumptions
	Changing views or perspectives
	Two-way learning
System Impacts	
	Creation of new relationships
	Changes in networks/boundaries
	Creation of new roles/practices
	Alignment of system parts
	Experimentation
	New mental models
	Identifying actions which are intended to have a systems impact
Connection to Systemic Problem	

The coding structure was informed by the theoretical insights of current systems thinking literature (Checkland and Poulter 2010; Wadsworth^2010^; Midgley 2006). The aim was to analyse the research data (transcripts and templates), to identify the learning which the community project team had gained from the process, in particular, the learning which had occurred at a ‘systemic’ level, or a higher level of abstraction (double loop learning) than learning simply about activities taking place within the system (single loop learning) (Jaaron and Backhouse 2017; Reynolds 2014).

The intention of introducing SAL as a complex intervention into the system, was to help practitioners identify and act on systemic problems that were affecting the implementation of the identified project. In comparison to standard health promotion activity, this approach is very different. The intervention point is practice based decision making. Not through prescriptive guidelines or manuals but rather opportunities to learn about the system and shift ‘mental models’ (Senge 2006) about what is possible. It seeks to create a more significant and enduring shift (even if small) by linking systems thinking expertise with real world practitioners’ expert knowledge of the local system.

## Results

Our proposition was that injecting systemic action learning (through the Prevention Tracker project) as a complex intervention would facilitate system level learning and enable local community teams to both learn and act on systemic problems through their day to day practice. Our results are promising. We gathered data about ongoing learning, insights and system impacts.

**Fig. 1:**
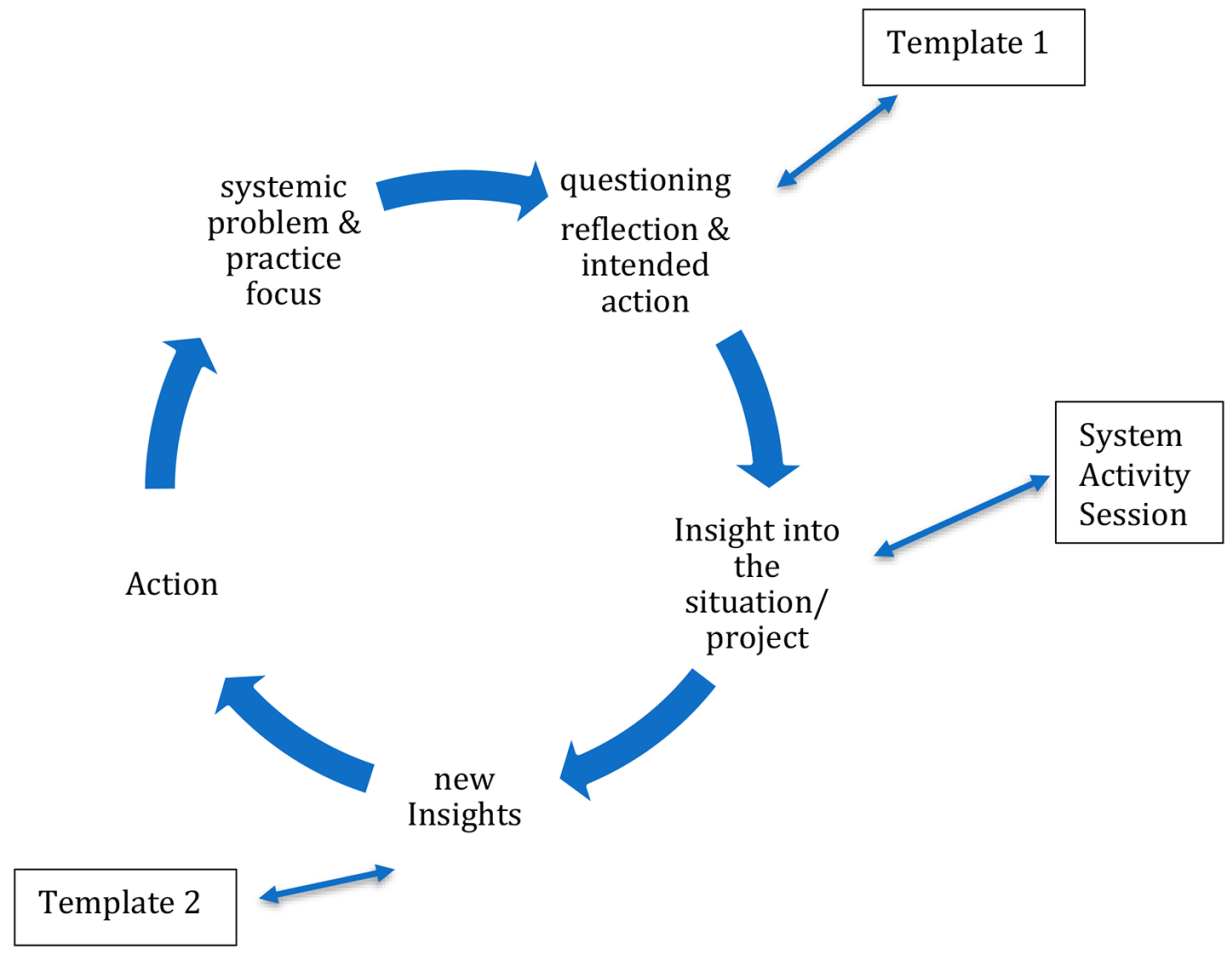
System action learning cycle in Prevention Tracker

The iterative nature of the SAL process (see Fig. 1) means that the results of our study take a circular, rather than linear pattern, whereby the learning at each step shapes and informs the subsequent steps, in an active sequence of feedback and response. The dynamic nature of the learning also means that new issues emerge while others become less important. For this reason, we present the findings of the SAL in two ways. First, we present a snapshot across three cycles of one of the System Action Learning cases (Case 2) according to the inter-relationship between the system activity sessions, insights gained and actions taken. We then describe each of the SAL stages, and examples from the cases. Where relevant, we include examples of how the data were coded. For clarity we have only included one code for each datum, although data may have been coded against a number of codes presented in Table 1.

### Cycles of System Action Learning

**Fig. 2:**
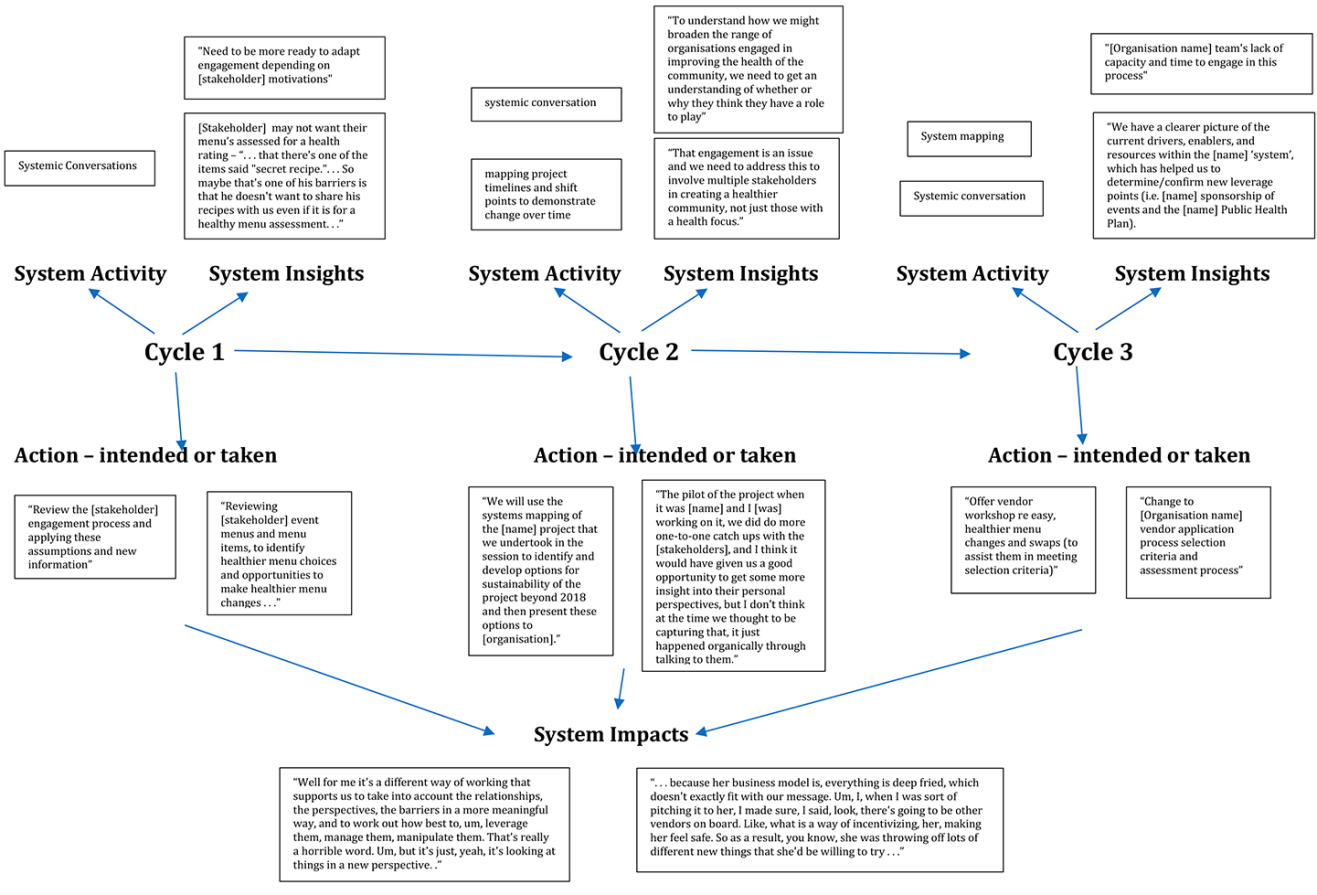
A snapshot of three cycles of system action learning

Figure 2 presents a snapshot of system activities, insights and actions across three cycles of systems action learning in Case 2. The case was a healthy eating initiative where the systemic problem/practice focus was the engagement of nontraditional actors in prevention efforts. In this case, food providers and the organization engaging their services. In each cycle we present the system activity that was undertaken, the type of system insights gained (via quotations) and the actions that were either taken or intended. These learnings are then taken into the next cycle and so on.

Figure 2 highlights the dynamic nature of learning through action over time. We now describe each of the System Action Learning phases (represented in Fig. 1) in greater detail.

#### Systemic Problem & Practice Focus

The starting point for each of the three SAL projects within Prevention Tracker was the identification of a systemic problem identified through a collaborative process between the researchers and stakeholders in the local community. This was facilitated by a systems thinking expert to help draw out a system level problem which might be hindering the way that chronic disease prevention activities were carried out in the community.

Each group of community partners identified a specific problem that was locally relevant to them, however the overarching key themes which appeared across communities in these systemic problem statements included issues of leadership, collaborative practice and partnering between organisations. The process of identifying the systemic problems are described elsewhere and were completed prior to the start of the SAL phase of the project (Riley et al. 2020). Each community project team then identified a practice focus, which was an existing prevention activity which was underway and indicative of the identified systemic problem. The practice focus would shape the systems activity sessions and subsequent cycles of reflection and action.

The following quotation is drawn from the third cycle of SAL, highlighting insights gained into the systemic problem of broadening the role of people and organizations involved in prevention. These insights emerged as the community team grappled with the evaluation of a partnership (practice focus)

*“Engagement is difficult if the perspectives, needs and assumptions of different stakeholders aren’t continually assessed and addressed to ensure stakeholders are ‘on the same page’ and working towards the same goal” (Case 3)*

#### Questioning & Reflection

Each of the projects had an existing meeting, governance and action structure within its community. The first step in the SAL cycle (as described above in the methods section) involved the local community project team using the SAL template to draw out systemic issues, for example issues pertaining to the role of the community project team at the systemic level, identifying perceptions or biases in the team’s thinking and developing insight into the problem being addressed. The template data informed the development of the SAL activity which was then undertaken with the research team and community partners.

One community team described (in Template 1 cycle 3) the issues they were grappling with at the time, in the following way

*“How do we make the intervention as simple and engaging as possible for [non traditional stakeholder group]?*

*How can we make the [name] program less resource/time intensive?” (Case 2, Code - Reflection on System Parts)*

This was followed up with “a ha” moments of making practical changes to the program to make it easier for stakeholders to be involved.

#### Insight Informs Actions

Participants in this phase of SAL used these insights to identify intentions to act on the system (above and beyond simply carrying out actions pertaining to their particular project). This action may be something they intended to do themselves to address the systemic problem (or practice focus), or action they anticipated others might take which would have an effect at the systems level.

In one case this resulted in a restructure of the next meeting that had been organized between two organisations partnering on a health promotion project. The community team had gained insight into the importance of reflecting on values, assumptions and differing perspectives when working across sectors and organisations. These insights informed the intended action – to restructure the next meeting, as the following quotation highlights.

*Speaker 3 (SAL participant): I’m just thinking in terms of how we will kind of set up this next meeting and how we might run it, as a bit of a discussion … [or] exploratory group, but I think we need to preface it in a way that shows them why we’re wanting to do what we’re about to do in terms of using these probing questions around what their values are, what their capacity is, etc.*

*Speaker 2 (SAL participant): Maybe we can preface it around that it’s just a reflection, given we’ve invested a lot of their time and our time in it around what we’ve done, what’s possible in the future, and you could also link in the [policy] stuff as well, just we’re really trying to make sure this fits in with your needs and your capacities and things. I don’t think you would really need it probed around, “We’re doing a systems exploration-“ (Case 2 code – Intention to Act)*.

In another example the community team gained insight into the strategic engagement of Stakeholders rather than blanket inclusion. In doing so, they began to foster new practices.

*we thought everyone had to be involved, and you know, if this particular [organization] wasn’t involved, it was a failure. We had done something wrong. Whereas, in actual fact it’s helped … me to step back and think, “Well actually no.“ (Case 2 code - New roles and practices)*.

#### Action

In between cycles of SAL, the teams in communities undertook action in accordance with their local project parameters and constraints. This could include project meetings, actions in the community and reflection within teams. Action undertaken by the community team within the community, was captured through the SAL templates, as well as in reflection during the next facilitated systems activity session.

In the following example, the community team were asked whether they had worked on an issue raised in the previous facilitated systems session, that of encouraging community ownership of a collaborative network.

*“That’s right. And we actually did talk about that at our last [collaborative network] meeting, um, that that would. .. be a real focus, even though we, you know, had the data and we had our Community Plan” (Case 1 code - Action Undertaken by SAL Team)*

The community team went on to describe how the desire for community ownership is reflected in a range of policies and intentions, but that the collaborative network may need to work differently in order to bring this about.

#### Analyse Outcomes

Community action was then followed up by the next cycle of SAL, in which reflection on action was undertaken and successful examples of action at the systemic level were identified.

In the following example the community team continued to work with the systems tools and ideas applied in the system activity session.

*After our last session with you guys we went away and did some more kind of activity work where we mapped those key project shift points for the project. And then, [we], mapped out our assumptions or learnings at each kind of shift point stage and what we did to address those or how we responded to those assumptions or learnings. So that has been useful in terms of incorporating that information to the evaluation. (Case 2 code – Action undertaken by SAL Team)*

#### Identify New Insights

Helping the community project teams identify systemic actions naturally led to them identifying new insights into the systems as well.

The participant’s comments above was followed by the reflection below:

*we might be explaining [to our partners] some of the assumptions, plus things that we did, and what it’s made us realize about the system and what we can and cannot control or influence or what the best leverage points are that we’ve identified. (Case 2 code - Surfacing Assumptions)*

Another community participant also reflected on the way their team was working, and identified opportunities to improve integration of their activities within the community :

*I think that it’s also changing the way that we operate and … and look at things. Ah, it’s the whole … team, so, we just had a discussion this morning about, basically going back to basics, and really looking at how we operate and how we best integrate into the community. (Case 1 code - Creation of New Roles and Practices)*

#### Systemic Problem & Practice Focus

These insights could then be applied back to the systemic problem, to identify opportunities for small but sustainable shifts.

An example of this came from one of the projects which incorporated its primary work into an expansion of role by a partner organization, and then, by extension, to other communities in the local region, thus changing the nature of local relationships as well as the boundaries of the local prevention system.

*it’s all kind of linked: it’s kind of like [the partnership initiative] started as this pilot project working to build the capacity of [organization 1]. We’re transitioning out of that now so not providing as much intensive support to [organization 1]. Setting it up for sustainability so it can do best practice health promotion, preventative health by their [policy] then [organization 2] is going to expand that support out to other [local] governments in the region using [the partnership initiative] as a kind of learning platform. So the learnings that we do gain through [the partnership initiative] and the Prevention Tracker work around evaluation will then I guess be applying where we can to [organization 1’s policy] but also the support that we provide in the future to other [local] governments in the region in terms of their … planning and evaluation. (Case 3 code - Reflection on Boundaries)*

## Discussion

For system action learning to take hold, practitioners within the community must be open and receptive to a new way of working, which can be very challenging. At the same time, those delivering the intervention must be flexible and open, willing to listen and adapt to the local environment and maintain focus on the systemic level while fighting the tendency to drift back towards the concrete level of the problem at hand. The example of cycles of system action learning presented in Fig. 2 highlights the type of system insights that can be gained through such processes. Each of the sessions surfaced new insights and actions at a system level. Through the cycles, the community team came to realize the importance of surfacing assumptions and drawing other perspectives into their learning. In addition, they committed to ensuring these learnings were shared with others in future meetings. In this way, their learning about the system was translated directly into practice. As a result, new practices, mental models and relationships became a part of the system of interest. These findings highlight the interconnected and relational nature of both gaining and applying system insights in practice. While much has been written about the importance of relationships in community-based prevention efforts (Trickett et al. 2011) including qualities of relationships such as trust (Bagnall et al. 2019), less attention has been paid to the deeply entangled nature of relationships, learning and action. Our findings suggest that while it is possible to observe key concepts, such as the creation of new relationships in practice data, the greatest insights may come from understanding how these concepts interact with each other through cycles of inquiry and over time.

Conceptualizing systems action learning as an intervention is challenging because few organisations within communities are funded or rewarded to work at this level. The overarching prevention system as it exists in Australia is fragmented and multi-sectoral/ multi-dimensional. No single bureaucratic home oversees prevention activities and the result is a patchwork of interventions and activities across public health, and many other sectors (including those at federal, state, local, non-government and community level). Therefore it requires a leap of faith from practitioners to commit the time and energy to a process which has often been compared to the actions of turning a huge ship by moving a small rudder (Senge 2006). While the overall benefits of undertaking systems change (for example, to work collaboratively to address chronic disease) may be more effective and more durable than single-action interventions (Waterlander et al. 2021), funding, time and governance restrictions tend to favour short-term, single-action interventions with measurable outcomes over longer term, higher level interventions with less predictable systemic impacts (Hopkins et al. 2021). In this regard, system action learning as an intervention may be better aligned with continuous quality improvement, where actions become the focus of reflection and inquiry and are embedded in organizational settings. Systems change is grounded in the day-to-day practices in organisations and collaborations. SAL is a mechanism for that to shift and change, influencing both the micro system of how the organization operates (practicing systems) as well as impact on their action and engagement in the system.Our analysis of system action learning in Prevention Tracker highlighted the importance of cycles of inquiry as practitioners learn over time and build knowledge in and through their action (Checkland and Poulter 2010). The emphasis on decision making in an immediate sense ensured the relevance of the ‘systems’ ideas to practice. Our ability to identify and describe learning processes (via codes such as double loop learning) linked to systemic ideas and systems change, suggests that it is possible to surface otherwise invisible aspects of system practice. In fact, it may be in the ‘private contexts of practice’ (Riley and Hawe 2009) rather than formalized training that systemic ideas take hold. This may go some way to explain Bensberg’s findings from the Healthy Together Victoria initiative (Bensberg 2021).

Our ‘cycle of inquiry’ is similar to others used in action research, action learning and improvement science (Foster-Fishman and Watson 2012; Wadsworth 2010, Taylor et al. 2014). All are designed to embed inquiry into practice. Similarly to Foster-Fishman and Watson (2012), we endeavoured to centre the inquiry process around ‘system’ insights. This required expert systems facilitation to enable real time exploration of practice decisions and their relationship to systems problems. The importance of systemic expertise has also been noted by others drawing attention to the effect of a systemic lens on implementation of initiatives (Pescud et al. 2021).

Despite the multitude of theoretical systems approaches which have been identified in the existing literature, comparatively few real world interventions have been able to study the cycle of systems action learning in practice, and to observe examples of systemic change in research data. Prevention Tracker offered a unique opportunity to collaborate between researchers and community-based practitioners to undertake systems action learning as well as scrutinize collected data for evidence of systemic change. Developing a thematic code book which was nuanced enough to identify small examples of systemic change in action, thinking, and learning was challenging. Coding required a deep understanding of each of the cases to interpret the data and this may not always be possible. However, the value in pursuing this inquiry lies in making visible and measuring otherwise invisible aspects of the process of change within complex systems. We invite others to apply, refine and strengthen the veracity of our coding scheme, including other forms of coding, which may yield additional insights.

## Conclusion

Designing and implementing complex, systems level interventions to address wicked problems lags behind work to identify and unpack those problems. Prevention Tracker offered an opportunity to guide, observe and demonstrate system level change in local community chronic disease prevention activities. The identification of systemic problems within community prevention systems, followed by facilitated learning about the problem and iterative cycles of action and reflection enabled the three Prevention Tracker SAL projects to surface assumptions, re-orient action and create new relationships and boundaries within and between complex systems. Theoretically informed analysis of action learning data in the form of transcripts of facilitated systems activity sessions and reflective templates from project meetings enabled the research team to identify intervention points at which the local community project teams were working, not just to deliver their immediate prevention project, but to work more systemically to address problems which inhibit more effective work across the entire prevention system.

The complexity of systemic change and the required time frames to observe such change don’t lend themselves neatly to the timeframes of research funding. Much work remains to be done to continue tracking the cumulative small shifts at the systems level which could lead to more significant and sustainable action in addressing complex public health problems into the future.

## Data Availability

The data from this study is not publicly available.
